# Supplementary material for: Methylene blue accelerates liquid-to-gel transition of tau condensates impacting tau function and pathology
Source: Nat Commun. 2023 Sep 6;14:5444. doi: 10.1038/s41467-023-41241-6 (PMC10482834; doi:10.1038/s41467-023-41241-6)
Supplement: Supplementary file 1 — Supplementary Information [file 41467_2023_41241_MOESM1_ESM.pdf]

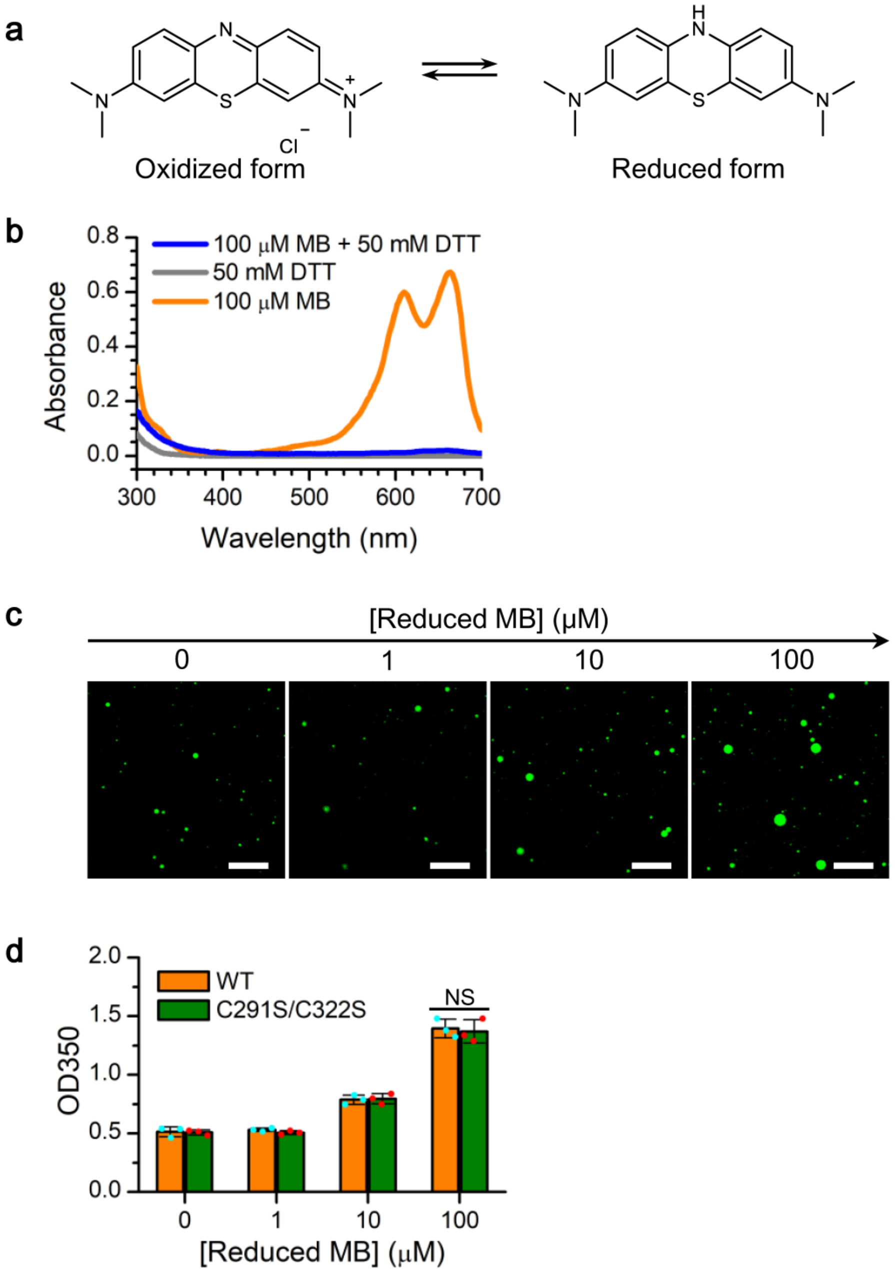

Supplementary Figure 1. The influence of the MB redox state on tau phase separation. **a** Transition between the oxidized form and the reduced form of MB. **b** UV-vis spectra showing that MB was reduced upon DTT treatment in 50 mM Tris (pH 7.4). **c** Representative fluorescence microscopy images of tau droplets (5 μM) with different concentrations of DTT-reduced MB, as indicated, in 50 mM Tris (pH 7.4) with 5% PEG8000. Scale bars, 10 μm. **d** Turbidity of tau solution (5 μM) in the presence of different concentrations of DTT-reduced MB, as indicated, in 50 mM Tris (pH 7.4) with 5% PEG8000. Data are presented as mean values +/− SD of three experiments. Significance levels were determined by unpaired two-sided Student's *t*-test. NS, non-significant. Source data are provided as a Source Data file.

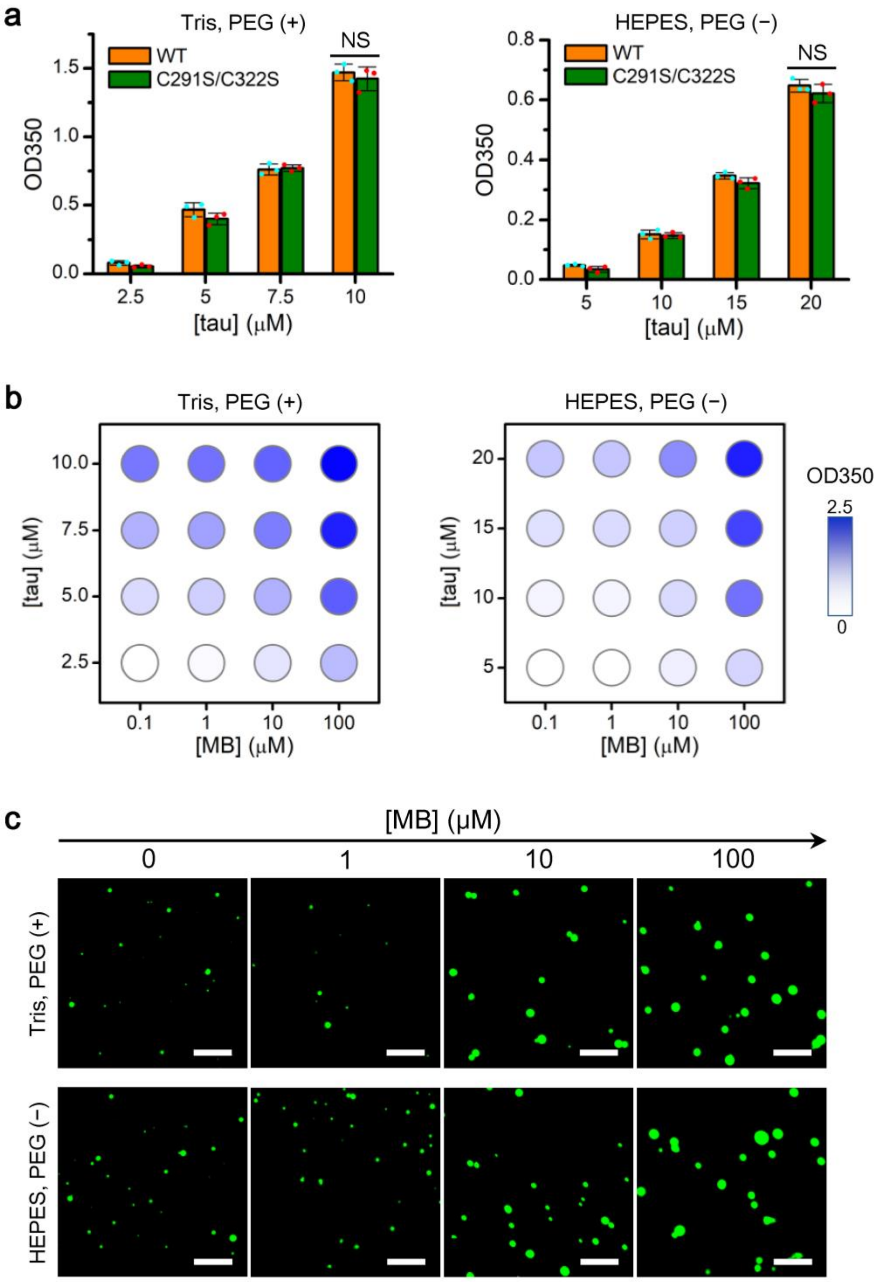

Supplementary Figure 2. The effect of MB on $tau_{C291S/C322S}$ phase separation. **a** Turbidity of tau solution measured at different protein concentrations in 50 mM Tris (pH 7.4) with 5% PEG8000 or 20 mM HEPES (pH 7.4) without PEG8000. Data are presented as mean values +/− SD of three experiments. Significance levels were determined by unpaired two-sided Student's *t*-test. NS, non-significant. **b** Turbidity of $tau_{C291S/C322S}$ solution measured at different $tau_{C291S/C322S}$ and MB concentrations, as indicated, in 50 mM Tris (pH 7.4) with 5% PEG8000 or in 20 mM HEPES (pH 7.4) without PEG8000. **c** Representative fluorescence microscopy images of $tau_{C291S/C322S}$ droplets with different MB concentrations. The concentrations of $tau_{C291S/C322S}$ were 5 μM and 20 μM in Tris buffer and HEPES buffer, respectively. Scale bars, 10 μm. Source data are provided as a Source Data file.

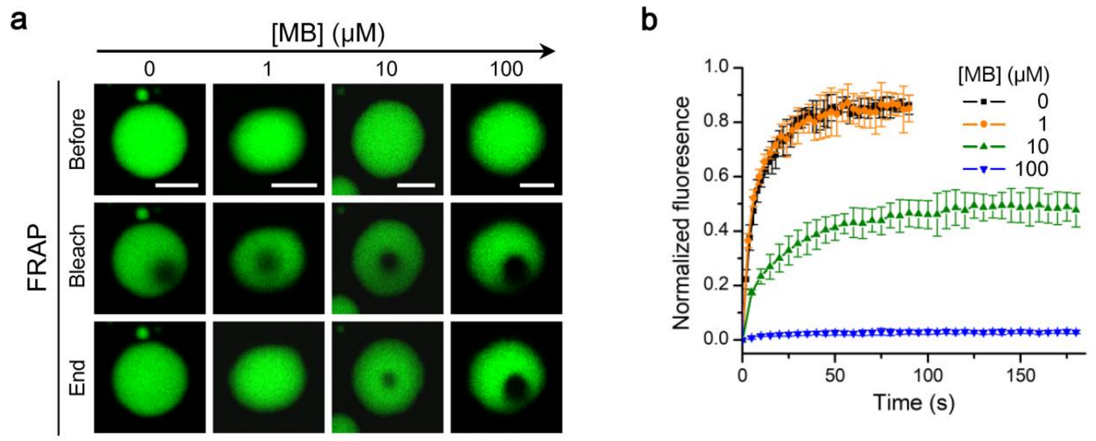

Supplementary Figure 3. FRAP experiments of WT tau droplets in Tris buffer. **a** Representative FRAP images of tau droplets formed by 10 μM tau with different concentrations of MB in 50 mM Tris (pH 7.4) with 5% PEG8000. Scale bars, 5 μm. **b** Quantified fluorescence intensity of the FRAP experiments. Data are presented as mean values +/− SD of three experiments. Source data are provided as a Source Data file.

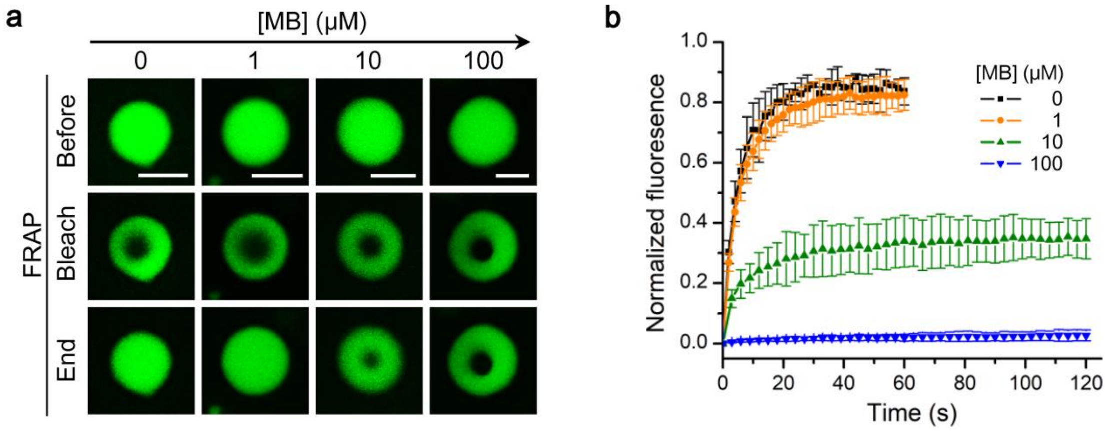

Supplementary Figure 4. FRAP experiments of tau$_{C291S/C322S}$ droplets in Tris buffer. **a** Representative FRAP images of tau droplets formed by 10 μM tau$_{C291S/C322S}$ in 50 mM Tris (pH 7.4) with 5% PEG8000 and different concentrations of MB. Scale bars, 5 μm. **b** Quantified fluorescence intensity of the FRAP experiments. Data are presented as mean values +/− SD of three experiments. Source data are provided as a Source Data file.

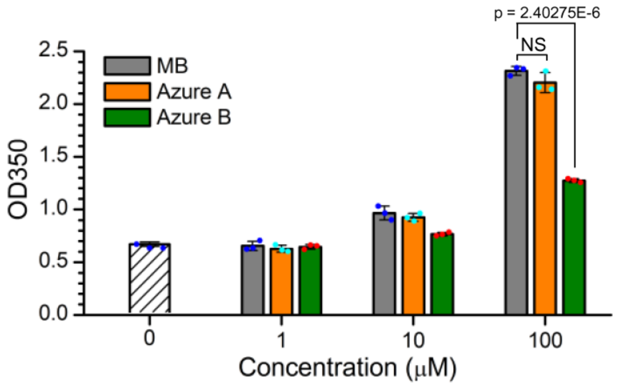

Supplementary Figure 5. Turbidity of 5 μM WT tau solution with different concentrations of azure A and azure B, as indicated, in 20 HEPES (pH 7.4) without PEG8000. Data are presented as mean values +/− SD of three experiments. Significance levels were determined by unpaired two-sided Student's *t*-test. NS, non-significant. Source data are provided as a Source Data file.

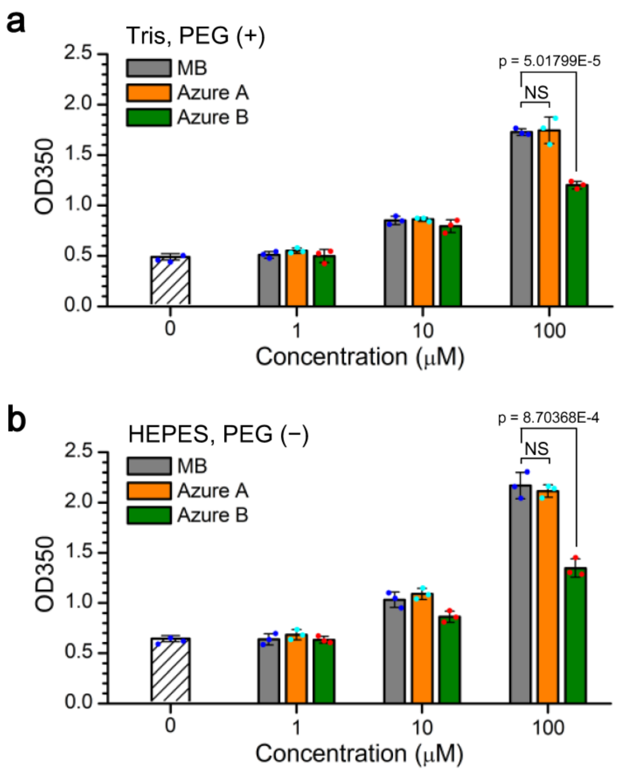

Supplementary Figure 6. Turbidity of 5 μM tau$_{C291S/C322S}$ solution with different concentrations of azure A and azure B, as indicated, in 50 mM Tris (pH 7.4) with 5% PEG8000 (**a**) or 20 mM HEPES (pH 7.4) without PEG8000 (**b**). Data are presented as mean values +/− SD of three experiments. Significance levels were determined by unpaired two-sided Student's *t*-test. NS, non-significant. Source data are provided as a Source Data file.

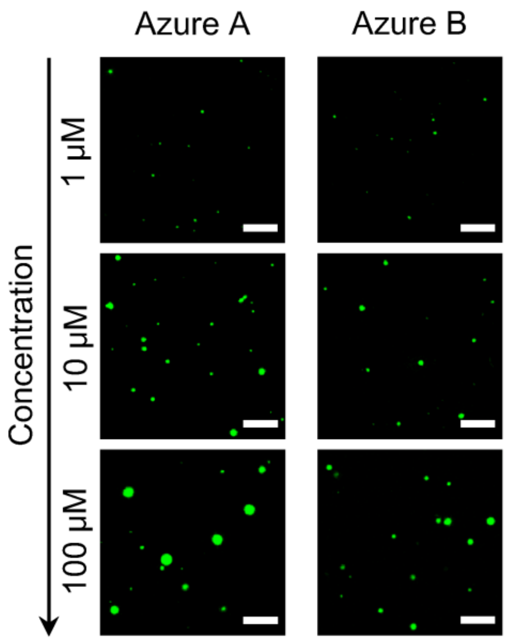

Supplementary Figure 7. Representative fluorescence microscopy images of tau droplets formed by 5 μM WT tau in 50 mM Tris (pH 7.4) with 5% PEG8000 and different concentrations of azure A and azure B. Scale bars, 10 μm.

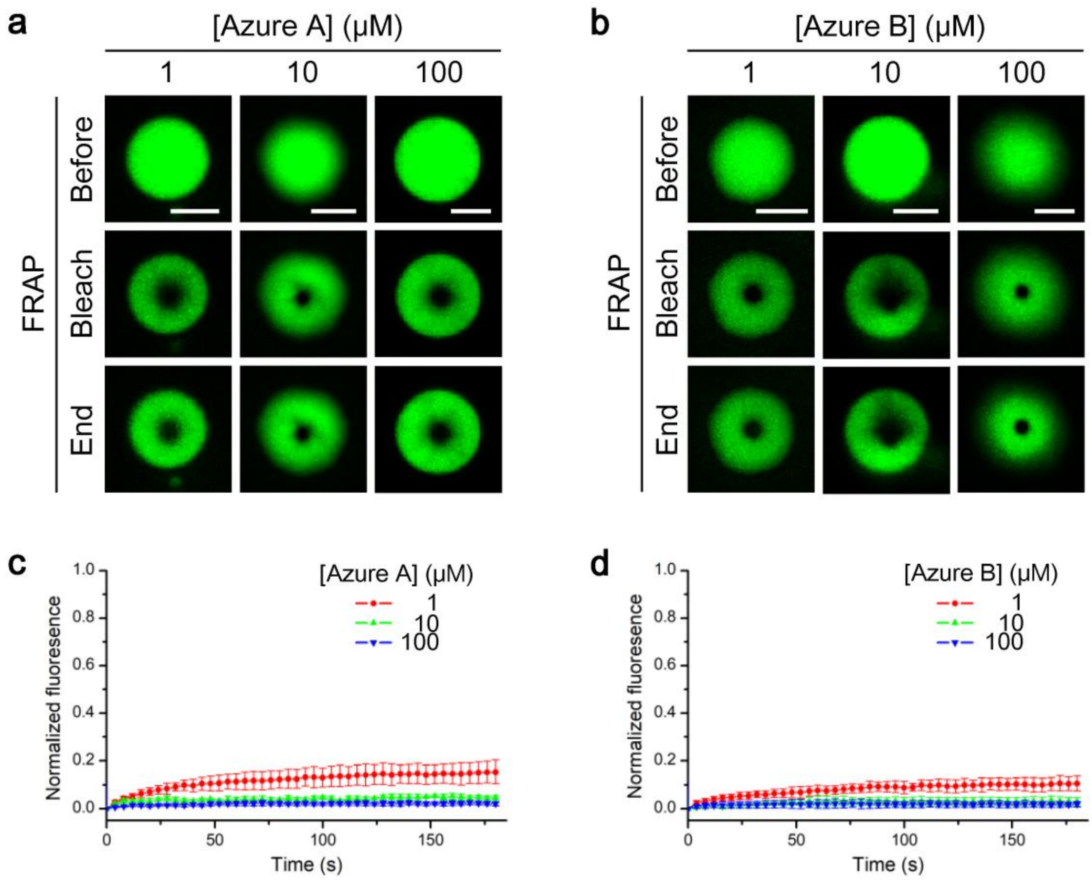

Supplementary Figure 8. FRAP experiments of tau droplets with different concentrations of azure A and azure B. **a, b** Representative FRAP images of tau droplets formed by 10 μM WT tau in 50 mM Tris (pH 7.4) with 5% PEG8000 and different concentrations of azure A and azure B, as indicated. Scale bars, 5 μm. **c, d** Quantified fluorescence intensity of the FRAP experiments. Data are presented as mean values +/− SD of three experiments. Source data are provided as a Source Data file.

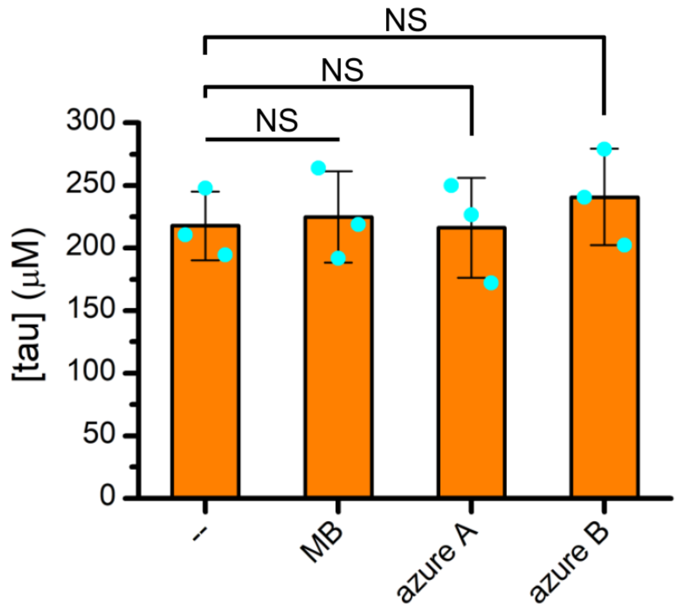

Supplementary Figure 9. Concentration of tau in the dense phase. Droplets were formed by incubating 5 μM WT tau in the presence or absence of 100 μM MB, azure A, or azure B in 50 mM Tris (pH 7.4) with 5% PEG8000. Data are presented as mean values +/− SD of three experiments. Significance levels were determined by unpaired two-sided Student's *t*-test. NS, non-significant. Source data are provided as a Source Data file.

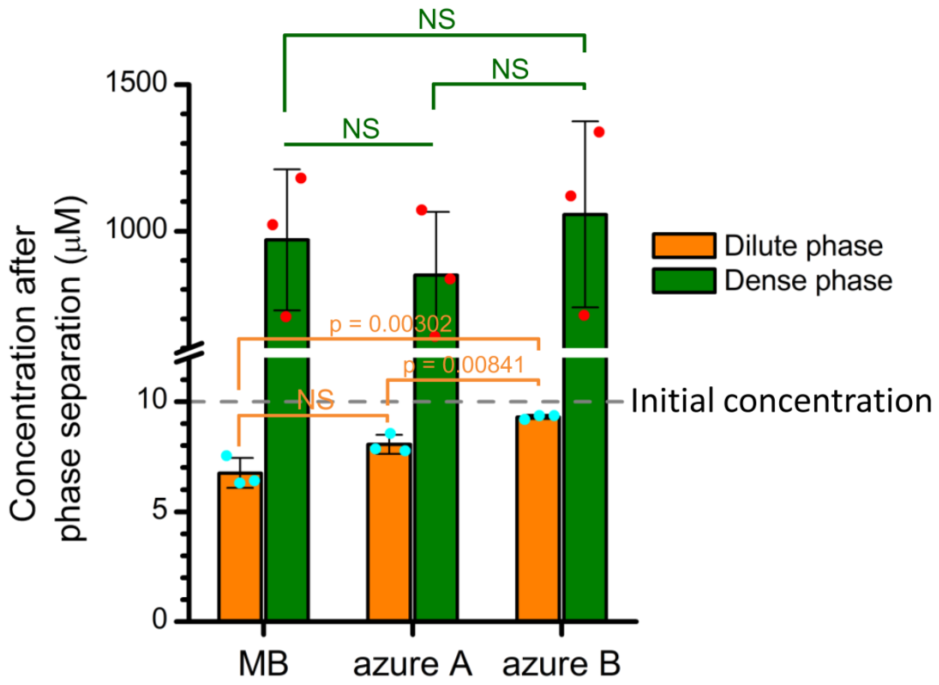

Supplementary Figure 10. Concentrations of compounds in the dilute phase and dense phase. Droplets were formed by incubating 5 μM WT tau with 10 μM MB, azure A, or azure B in 50 mM Tris (pH 7.4) with 5% PEG8000. Data are presented as mean values +/− SD of three experiments. Significance levels were determined by unpaired two-sided Student's *t*-test. NS, non-significant. Source data are provided as a Source Data file.

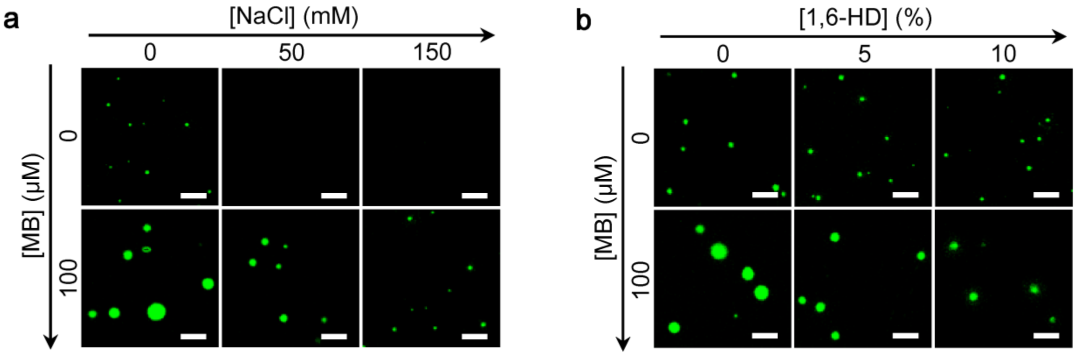

Supplementary Figure 11. Representative fluorescence microscopy images of WT tau droplets (5 μM) with different concentrations of NaCl (**a**) and 1,6-HD (**b**) in 50 mM Tris (pH 7.4) with 5% PEG8000. Scale bars, 5 μm.

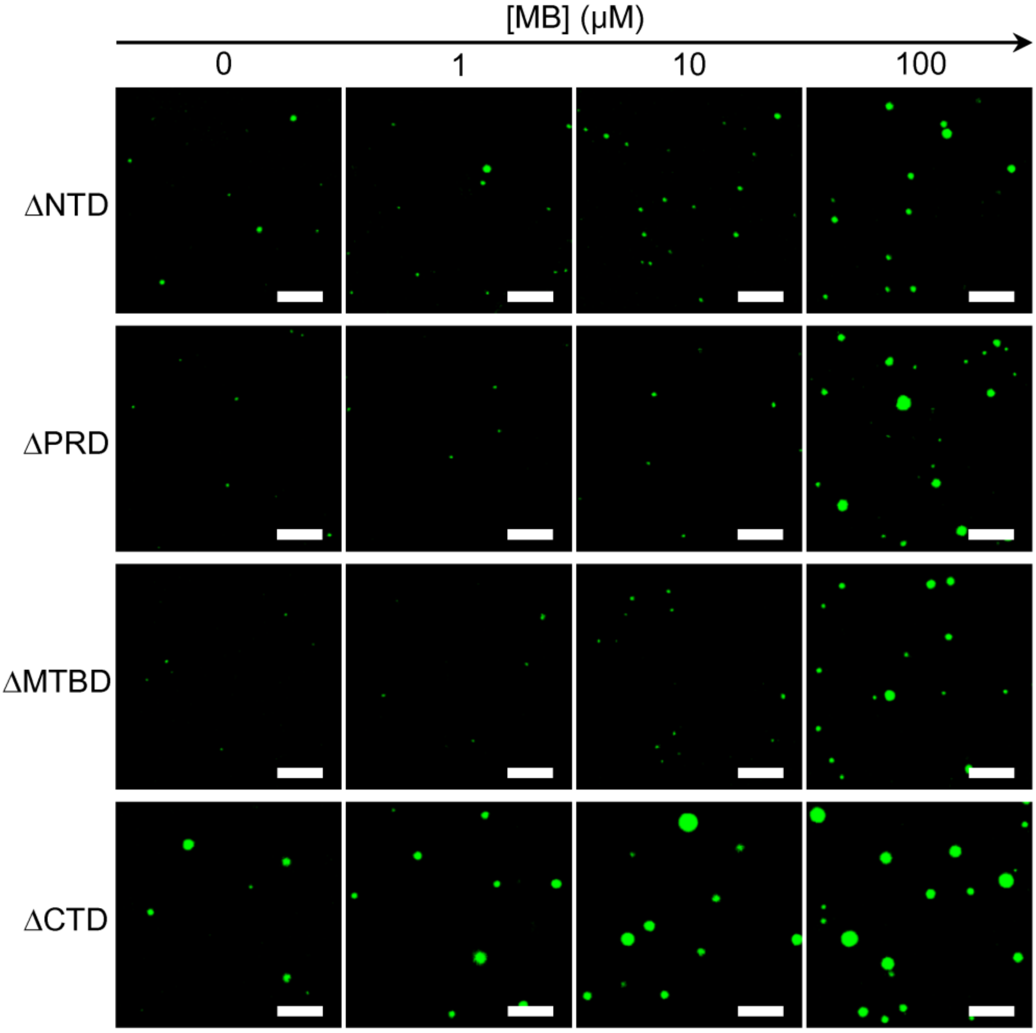

Supplementary Figure 12. Representative fluorescence microscopy images of droplets formed by tau deletion variants (5 μM) in 50 mM Tris (pH 7.4) with 5% PEG8000 in the presence of different concentrations of MB. Scale bars, 10 μm.

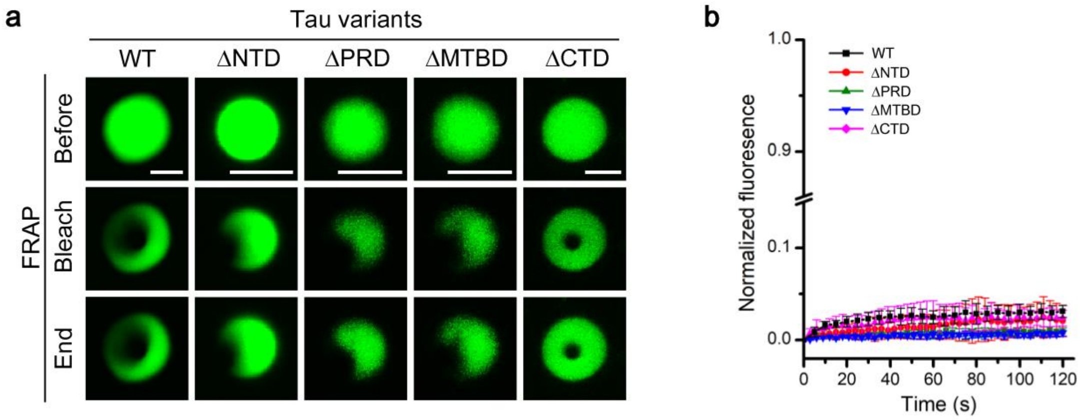

Supplementary Figure 13. FRAP experiments for tau deletion variants. **a** Representative FRAP images of tau droplets formed by tau variants in 50 mM Tris (pH 7.4) with 5% PEG8000 and 100 μM MB. The concentrations were 10 μM for WT tau and ΔCTD and 20 μM for ΔNTD, ΔPRD, and ΔMTBD. Scale bars, 5 μm. **b** Quantified fluorescence intensity of the FRAP experiments. Data are presented as mean values +/− SD of three experiments. Source data are provided as a Source Data file.

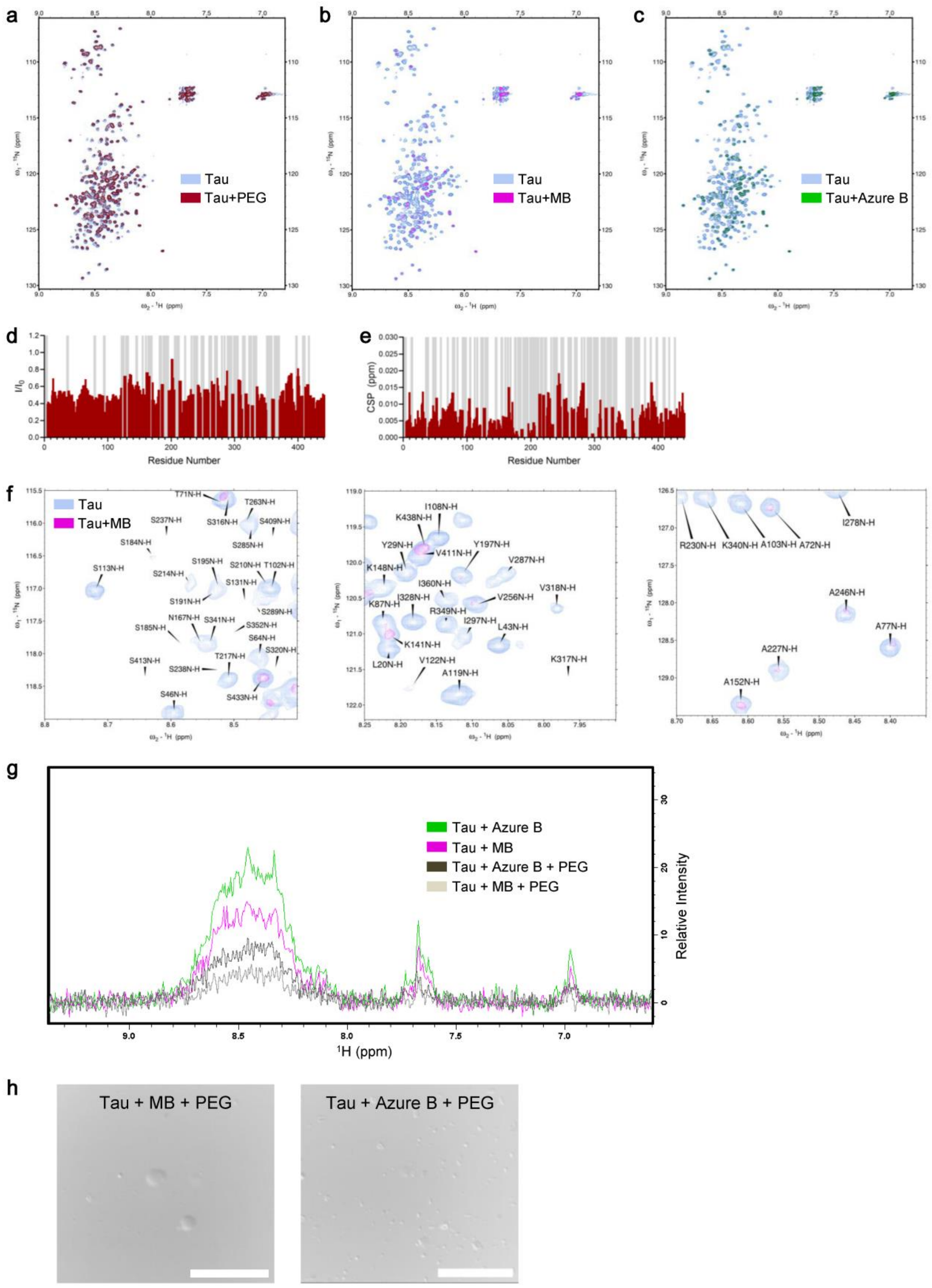

Supplementary Figure 14. Tau NMR spectral changes corresponding to LLPS conditions. **a-c** Overlay of 2D ^{1}H-^{15}N SOFAST-HMQC spectra of tau with and without 5% PEG8000 (**a**), and with and without 360 µM MB (**b**) or azure B (**c**). **d** Peak amplitude ratios (I/I_0) calculated from the 2D ^{1}H-^{15}N SOFAST-HMQC spectra of tau with and without 5% PEG8000. **e** Chemical shift perturbations (CSP) calculated from the 2D ^{1}H-^{15}N SOFAST-HMQC spectra of tau with and without 5% PEG8000.

Residues omitted from the I/I_0 and CSP analyses due to lack of reliable peak assignment are designated as grey bars. **f** Selected regions of the ^{1}H-^{15}N SOFAST-HMQC spectra showing NMR signal attenuation of tau residues corresponding to the NTD, PRD, MTBD, and CTD of tau upon MB addition. **g** 1D projections (^{1}H dimension) of the 2D ^{1}H-^{15}N SOFAST-HMQC spectra of tau in the presence and absence of 360 μM MB or 360 μM azure B, with and without 5% PEG8000. **h** DIC micrographs of the NMR samples in the presence of MB or azure B and 5% PEG8000. Scale bars, 20 µm. 2D ^{1}H-^{15}N SOFAST-HMQC spectra were collected with 18 μM tau in 50 mM Tris (pH 7.4) at 5 ℃. Source data are provided as a Source Data file.

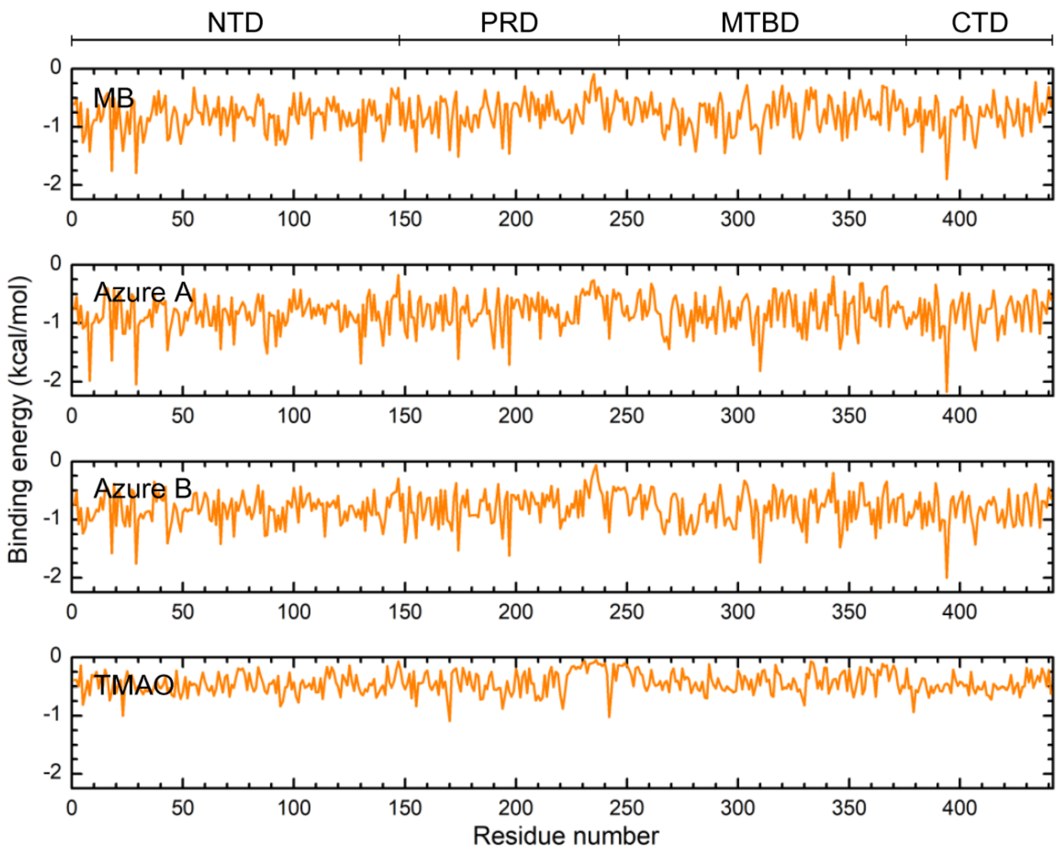

Supplementary Figure 15. Tau residue-ligand binding profiles calculated using 1000 docking poses for MB, azure A, azure B, and TMAO. Source data are provided as a Source Data file.

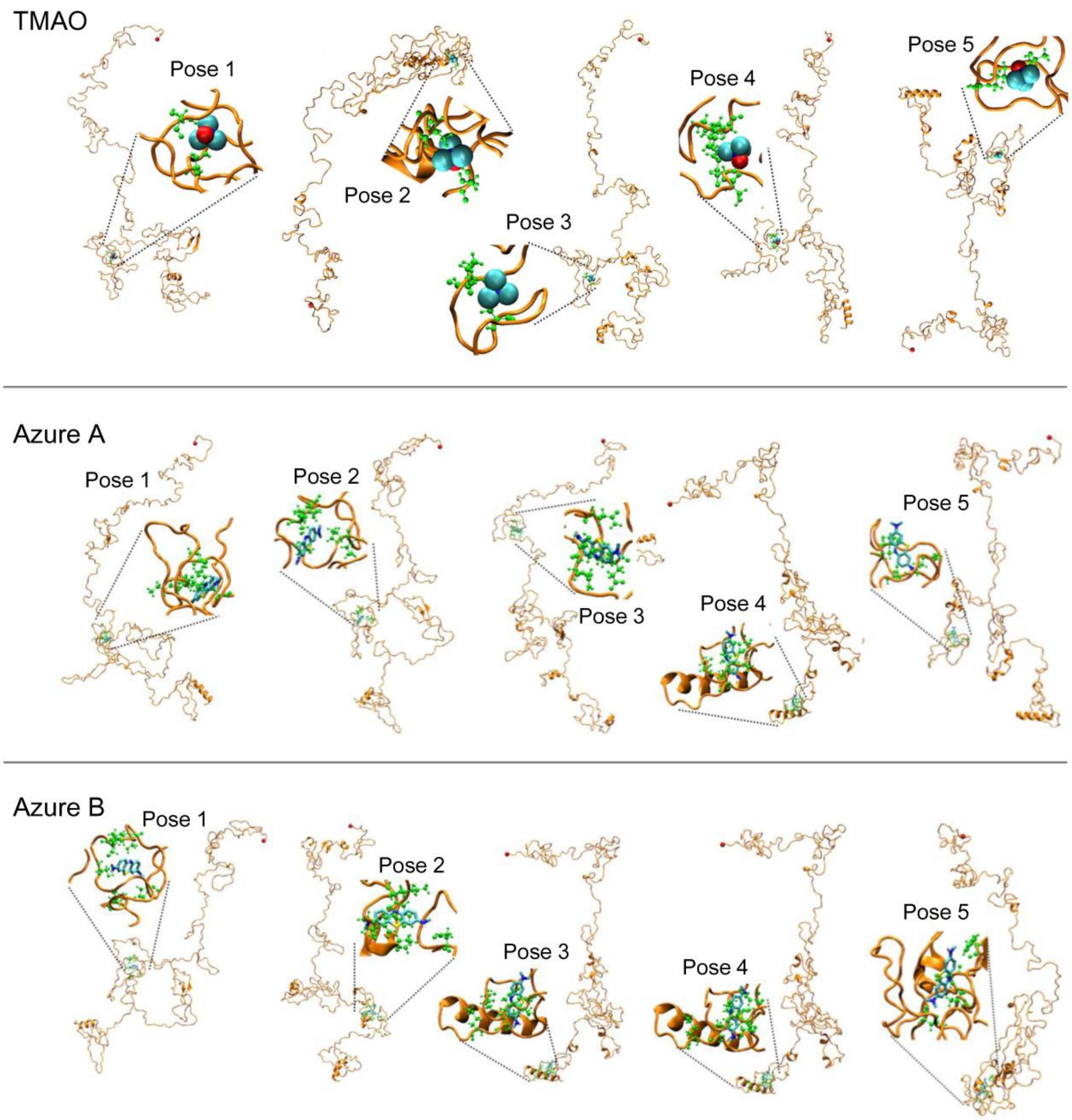

Supplementary Figure 16. The top 5 docking poses of azure A, azure B, and TMAO binding to WT tau. The N-terminus of tau is shown as a red ball. The residues which have strong interaction with the ligand are shown as green balls and sticks.

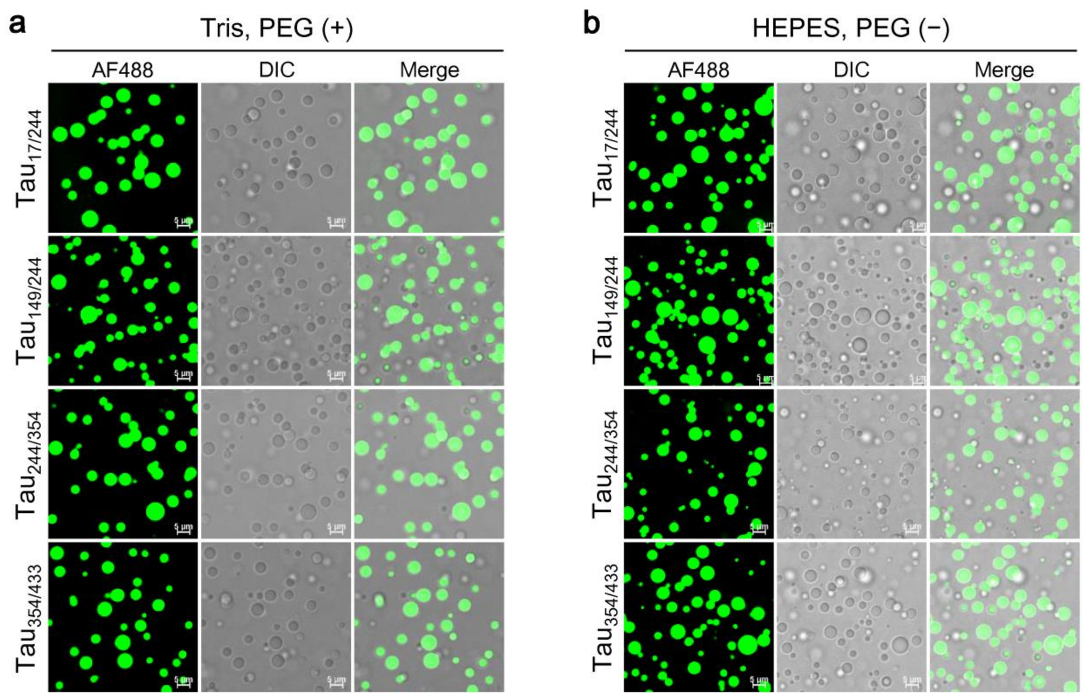

Supplementary Figure 17. Representative fluorescence microscopy images of droplets formed by WT tau doped with 10% AF350/AF488-labeled tau variants in 50 mM Tris (pH 7.4) with 5% PEG8000 (**a**) or 20 mM HEPES (pH 7.4) without PEG8000 (**b**) in the presence of 100 μM MB, indicating that the AF350/AF488-labeled tau was enriched in the dense phase. Scale bars, 5 μm.

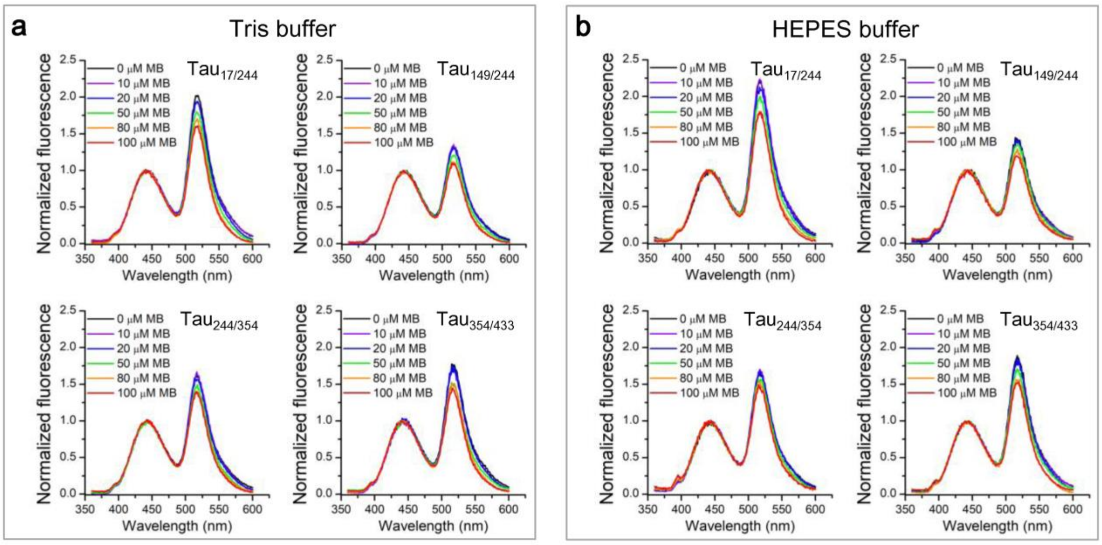

Supplementary Figure 18. FRET measurements of AF350/AF488-labeled tau variants under non-phase-separating conditions. FRET spectra of 0.2 μM AF350/AF488-labeled tau variants without unlabeled tau at different MB concentrations, as indicated, in 50 mM Tris (pH 7.4) without PEG8000 (**a**) or 20 mM HEPES (pH 7.4) without PEG8000 (**b**). Source data are provided as a Source Data file.

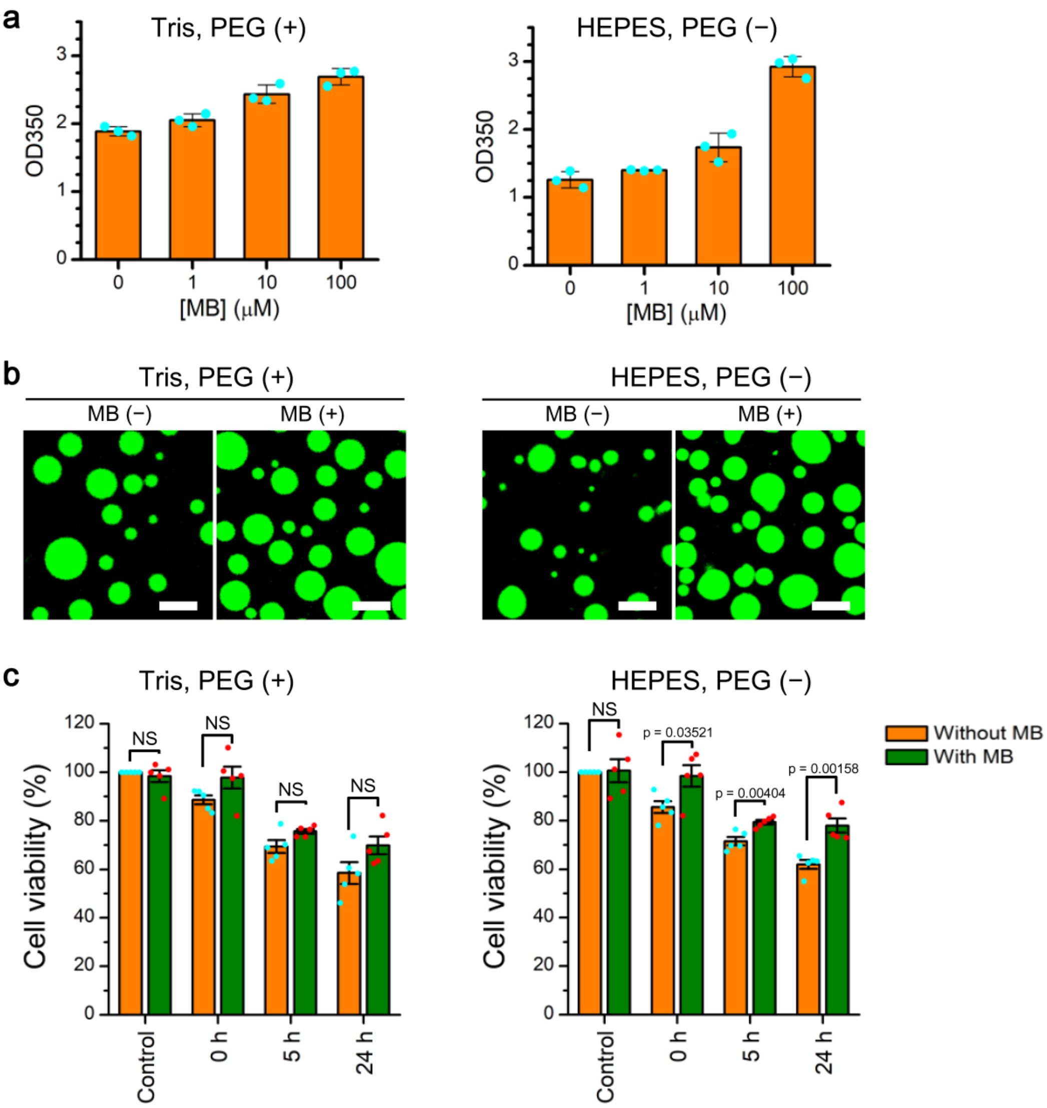

Supplementary Figure 19. The effects of MB on the phase separation and cytotoxicity of WT tau in the presence of heparin. **a** Turbidity of tau solution at different concentrations of MB, as indicated. Data are presented as mean values +/− SD of three experiments. **b** Representative fluorescence microscopy images of tau droplets in the presence or absence of 100 μM MB. Scale bars, 10 μm. **c** MB reduced tau cytotoxicity in the context of LLPS. SH-SY5Y cells were treated with tau droplets incubated with heparin for different times, with or without 100 μM MB. Viability was measured using an MTT assay. A solution of heparin without tau was used as a control. Data are presented as mean values +/− SE of five experiments. Significance levels were determined by unpaired two-sided Student's *t*-test. NS, non-significant. A solution of 10 μM tau in 50 mM Tris (pH 7.4) with 7.5% PEG8000 or 15 μM tau in 20 mM HEPES (pH 7.4) without PEG8000 was incubated with 5 μM heparin and different concentrations of MB, as indicated. Source data are provided as a Source Data file.

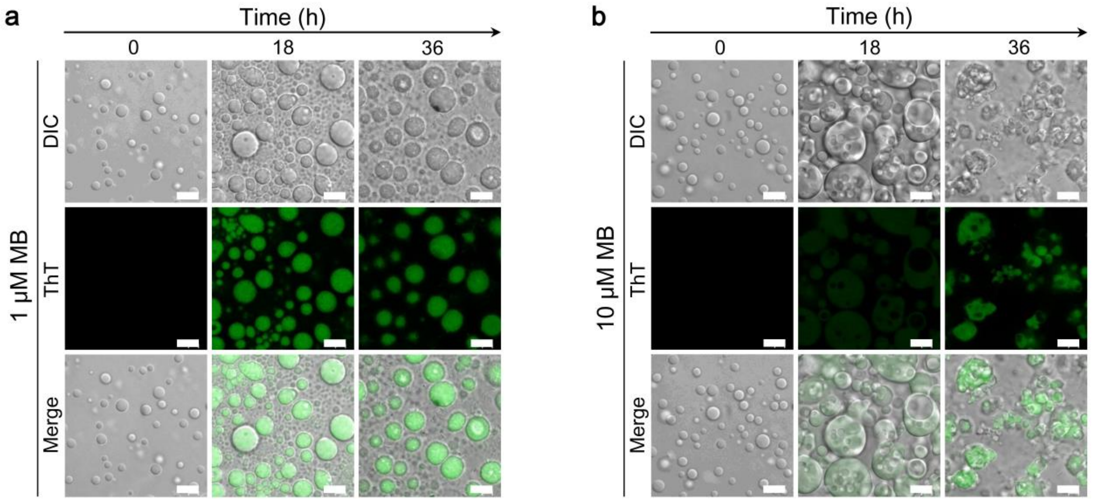

Supplementary Figure 20. Representative microscopy images of the growth of tau aggregates inside droplets in the presence of 1 μM (**a**) or 10 μM (**b**) MB in 50 mM Tris (pH 7.4) with 7.5% PEG8000 and 5 μM heparin. Scale bars, 10 μm.

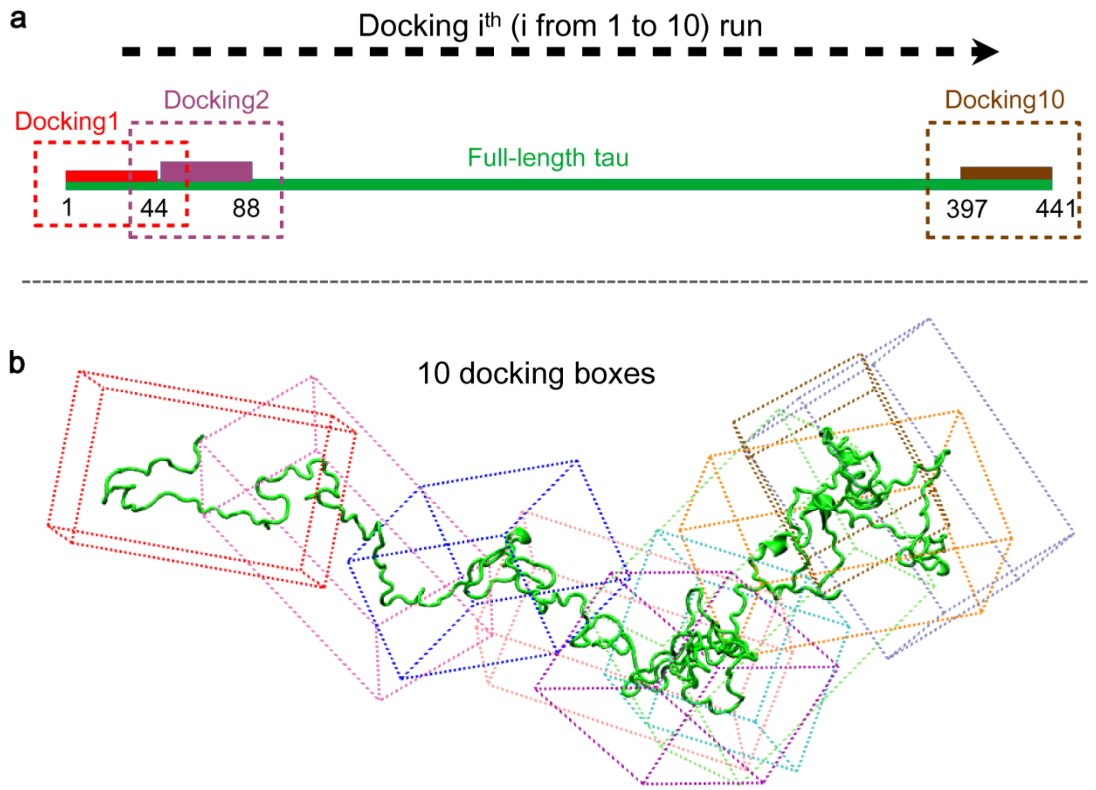

Supplementary Figure 21. Docking setup for studying ligand-tau interaction. **a** Full-length tau is equally divided into 10 fragments, the residues in each fragment range from $(i-1) \times 44 + 1$ to $i \times 44 + 1$. **b** The docking boxes of the 10 docking runs shown as cuboids.

Supplementary Table 1. Fitting equations of aspect ratio measurements and halftimes of fusing droplets in the presence of different MB concentrations.

[MB] (μM)	Fitting equation	Halftime (min)
0	$Y = 1.6544 - 0.67351 \exp(-0.01266\, x)$	94
1	$Y = 2.4855 - 1.4583 \exp(-0.00403\, x)$	85
10	$Y = 1.9209 - 0.90825 \exp(-0.08609\, x)$	8
100	$Y = 1.9277 - 0.91769 \exp(-0.10806\, x)$	6

Aspect ratios (ARs) were fitted using the following equation:
$Y = Y_0 + A\cdot\exp(-\tau^{-1}\, x)$
where, Y is the AR at time x, A is a constant, (Y_0+A) is the initial AR, and τ is the time constant. The maximum AR is 1.9 for all conditions, considering that the AR for 0 and 1 μM MB will also reach around 1.9 if the aging time is long enough. The data fitting also included the point (0, 1) as the initial, i.e. it is considered that at 0 time, all the droplets fuse to a perfect spherical shape with AR = 1.0.

Supplementary Table 2. Fluorescence lifetime of AF488 in AF488-labeled tau under different conditions.

Buffer	Component	Fluorescence lifetime (ns)	Sample state
50 mM Tris, pH 7.4	AF488-tau	4.0	No LLPS
	AF488-tau + 100 µM MB	4.0	No LLPS
	AF488-tau + 10 µM tau + 5% PEG8000	3.7	LLPS
	AF488-tau + 10 µM tau + 5% PEG8000 + 100 µM MB	3.7	Strong LLPS
20 mM HEPES, pH 7.4	AF488-tau	3.6	No LLPS
	AF488-tau + 100 µM MB	3.7	No LLPS
	AF488-tau + 15 µM tau	3.9	Weak LLPS
	AF488-tau + 15 µM tau + 100 µM MB	3.8	Strong LLPS
